# Ozone Pollution: A Major Health Hazard Worldwide

**DOI:** 10.3389/fimmu.2019.02518

**Published:** 2019-10-31

**Authors:** Junfeng (Jim) Zhang, Yongjie Wei, Zhangfu Fang

**Affiliations:** ^1^Nicholas School of the Environment and Duke Global Health Institute, Duke University, Durham, NC, United States; ^2^Global Health Research Center, Duke Kunshan University, Kunshan, China; ^3^Guangzhou Institute of Respiratory Health, Guangzhou Medical University, Guangzhou, China; ^4^State Key Laboratory of Environmental Criteria and Risk Assessment & Environmental Standards Institute, Chinese Research Academy of Environmental Sciences, Beijing, China; ^5^Center for Global Health, School of Public Health, Nanjing Medical University, Nanjing, China

**Keywords:** ozone, climate change, air quality standards, cardiovascular health effects, respiratory health effects, mitigation strategies

## Abstract

Oxides of nitrogen (NO_x_) and volatile organic compounds (VOCs) released into the atmosphere can react in the presence of solar irradiation, leading to ozone formation in the troposphere. Historically, before clean air regulations were implemented to control NO_x_ and VOCs, ozone concentrations were high enough to exert acute effects such as eye and nose irritation, respiratory disease emergencies, and lung function impairment. At or above current regulatory standards, day-to-day variations in ozone concentrations have been positively associated with asthma incidence and daily non-accidental mortality rate. Emerging evidence has shown that both short-term and long-term exposures to ozone, at concentrations below the current regulatory standards, were associated with increased mortality due to respiratory and cardiovascular diseases. The pathophysiology to support the epidemiologic associations between mortality and morbidity and ozone centers at the chemical and toxicological property of ozone as a strong oxidant, being able to induce oxidative damages to cells and the lining fluids of the airways, and immune-inflammatory responses within and beyond the lung. These new findings add substantially to the existing challenges in controlling ozone pollution. For example, in the United States in 2016, 90% of non-compliance to the national ambient air quality standards was due to ozone whereas only 10% was due to particulate matter and other regulated pollutants. Climate change, through creating atmospheric conditions favoring ozone formation, has been and will continue to increase ozone concentrations in many parts of world. Worldwide, ozone is responsible for several hundreds of thousands of premature deaths and tens of millions of asthma-related emergency room visits annually. To combat ozone pollution globally, more aggressive reductions in fossil fuel consumption are needed to cut NO_x_ and VOCs as well as greenhouse gas emissions. Meanwhile, preventive and therapeutic strategies are needed to alleviate the detrimental effects of ozone especially in more susceptible individuals. Interventional trials in humans are needed to evaluate the efficacy of antioxidants and ozone-scavenging compounds that have shown promising results in animal studies.

## Sources and Chemistry

Ozone, the triplet oxygen (O_3_), is formed from the reaction between dioxygen (O_2_, the normal oxygen molecule) and a singlet oxygen (O, oxygen atom) in the presence of a third-body molecule able to absorb the heat of the reaction. The highly reactive and short-lived singlet oxygen (O) can be generated via the photolysis of nitrogen dioxide (NO_2_) or ionization of O_2_. Background ozone is present in both the stratosphere and the troposphere. Stratospheric ozone is concentrated in the tropopause (~between 8 and 15 km above the ground), a region that is called ozone layer. Stratospheric ozone is nicknamed “good” ozone, because the ozone layer plays a vital role in absorbing ultraviolet (UV-B) rays that are harmful to living beings on the earth. Since direct contact with ozone at the ground level can cause damages to living cells, organs, and species including humans, animals, and plants, tropospheric or ground-level ozone is nicknamed “bad” ozone.

**Figure 1:**
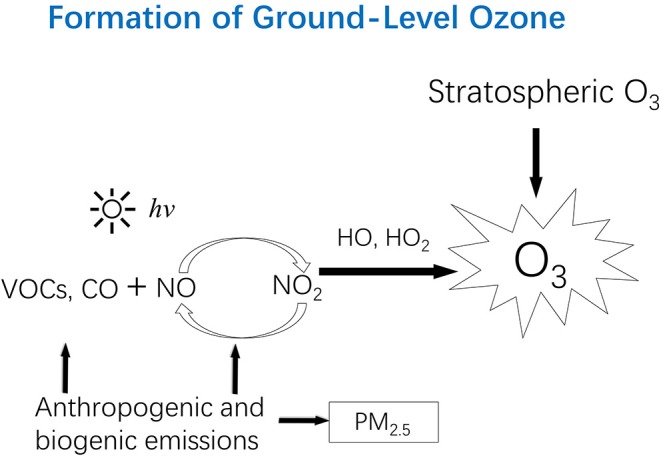
Ozone in the stratosphere can move downward to the troposphere, contributing to the “background” level of ground-level ozone. However, high levels of ozone in the troposphere are due to photochemical reactions involving volatile organic compounds (VOCs) and oxides of nitrogen (NO_x_: NO, and NO_2_). Anthropogenic emissions (e.g., fossil fuel combustion) are responsible for NO_x_ and mainly responsible for VOCs and CO. Trees also emit certain VOCs (e.g., isoprene). PM_2.5_ from primary emission sources can react with (consume) free radicals (e.g., HO_2_) responsible for ozone formation, which partly explains the observations in certain areas where ozone level increased while PM_2.5_ level decreased. *hv*, photon; VOCs, volatile organic compounds; CO, carbon monoxide; NO, nitric oxide; NO_2_, nitrogen dioxide; NO_x_, NO and NO_2_; HO, the hydroxyl radical; HO_2_, The hydroperoxy radical; PM_2.5_, Particulate matter with a diameter of 2.5 μm or less.

There is a natural influx of ozone from the stratosphere to the troposphere, peaking normally in the spring months when the vertical air movement reaches its maximum in the northern hemisphere. This influx contributes to background levels of ground-level ozone. The predominant source of tropospheric ozone, however, is the photochemical reactions involving volatile organic compounds (VOCs) and oxides of nitrogen (NO_x_), mainly comprised of NO_2_ and nitric oxide (NO). In the absence of or at very low concentrations of VOCs or carbon monoxide (CO), ozone reaches a steady-state concentration depending on solar intensity, ambient temperature, and the ratio of NO_2_ concentration to NO concentration. Under this condition, one NO_2_ molecule is converted via photolysis into one O_3_ molecule and one NO molecule; and ozone is, in turn, consumed by NO to regenerate a NO_2_ molecule. This cycle results in zero accumulation of ozone concentration. However, VOCs or CO participate in a series of complex photochemical reactions to produce free radicals that compete with ozone to react with NO. The net effects include the accumulation of ozone, the oxidation of VOCs into oxygenated organic compounds, and the formation of nitrogen-containing compound, and the oxidation of CO into CO_2_. Because many of the oxygenated and nitrogen-containing organic compounds are present in the condensed phase due to their low volatility, they are called secondary organic aerosols (SOAs). The whole mixture composed of ozone, SOAs, and their gaseous precursors is called photochemical smog. The production of ozone in the troposphere is depicted in Figure 1.

This ozone formation mechanism (Figure 1) explains why elevated ozone concentrations are found in an increasing number of places around the world where anthropogenic emissions of NO_x_, VOCs, and CO have been increasing. The combustion of fossil fuels occurs at a high temperature favorable for NO_x_ formation, and worldwide increases in fossil-fuel derived energy (for electricity generation, transportation, and household cooking and heating) are responsible for increasing emissions of NO_x_. Major anthropogenic sources of VOCs include vehicular exhaust, fugitive evaporation of gasoline and other gaseous fuels (e.g., natural gas and propane), biomass and fossil fuel combustion, and industrial solvent use. A recent study found that volatile chemicals released from consumer products (e.g., pesticides, coatings, printing inks, adhesives, cleaning agents, and personal care products) have emerged as a large urban source of VOCs (1). Natural vegetation emissions of certain VOCs (e.g., isoprene) also contribute to ozone formation especially at the regional scale (2–5).

Ozone formation depends on solar intensity that is directly associated with atmospheric temperature. Ironically, with a decrease in ambient concentrations of carbonaceous aerosols (e.g., soot), emitted from combustion of coal, diesel, and biomass, atmospheric visibility increases, and consequently solar intensity increases, favoring ozone formation. More importantly, particulate matter (e.g., particles with an aerodynamic diameter equal to or smaller than 2.5 μm, noted as PM_2.5_) can serve as a sink of free radicals responsible for ozone formation. A recent study showed that a 40% reduction in PM_2.5_ from 2013 to 2017 in the North China Plain was partly responsible for an increasing ozone trend (at 1–3 ppb per year) during the period of 2013–2017 observed in megacity clusters of eastern China (6).

## Impact of Climate Change on Ground-Level Ozone

Ozone itself is a greenhouse gas in the atmosphere. Hence, increasing ground-level ozone contributes to global warming. On the other hand, a warming climate favors the formation and accumulation of ozone in the atmosphere mainly through two physicochemical mechanisms. First, in certain parts of the world, a warming climate changes humidity and wind conditions, leading to decreases in the frequency of surface cyclones. The resulting more stagnant atmospheric condition decreases the dispersion of NO_x_ and VOCs and prolongs the time for the reactions to produce ozone. Second, ozone-forming reactions are typically enhanced by increased atmospheric temperatures. Based on these climate-induced changes in the atmospheric stability (air stagnation) and temperature, it is predicted that by the year 2050, warming alone may increase by 68% the number of ozone-standard exceedance days across the eastern United States (7). Another study predicted that changes in regional climate and globally enhanced ozone would increase ground-level ozone over most of the United States. More specially, it is predicted that the 95th percentile for daily 8-h maximum ozone would increase from 79 ppb in 2012 to 87 ppb in 2050 (8). Similarly, another predictive analysis, through integrating data from climate model outputs and historical meteorology and ozone observations across 19 urban communities in southeastern United States, estimated an increase of 0.43 ppb (95% CI: 0.14–0.75) in average ozone concentration during the 2040s compared to 2000 due to climate change alone (9).

Climate change can also prolong the ozone season. For example, high ozone concentrations usually occur in the summer in the United States. However, ozone during the fall reached the summer level in several Octobers in the 2000s and in 2010 over the southeastern United States. This was attributed to enhanced emissions of biogenic isoprene (a VOC precursor of ozone) from water-stressed plants under a drying and warming condition (10). This finding suggests that occurrences of a drying and warming fall in the future may lead to an extension of the ozone season from summer to fall in the regions with significant biogenic VOC emissions.

## Ambient Concentrations in Reference of Air Quality Standards

Ozone is in gas phase under typical atmospheric conditions (temperature and pressure) and is commonly measured as mixing ratio, i.e., parts per million (ppm) or parts per billion (ppb). At the standard conditions for temperature (25°C) and pressure (1 atmosphere), 1 ppb ozone equals 1.97 μg/m^3^. Based on its commonly recognized health effects, including causing breathing problems, triggering asthma attacks, reducing lung function, and increasing incidence of respiratory diseases, ozone is one of the regulated air pollutants in many countries and has a recommended limit by the World Health Organization (WHO). The current WHO Air Quality Guidelines for ambient (outdoor) ozone is 100 μg/m^3^ (~50 ppb) measured as 8-h maximum moving average within a day^1^. In the United States, the current National Ambient Air Quality Standards (NAAQS) for ozone include a 1-h standard (1-h maximum within a day) at 120 ppb and an 8-h standard (8-h daily max) at 70 ppb. The rationales for having two standards with different averaging times are as follows.

Historically, the NAAQS only had a 1-h standard, as a sharp peak of ozone concentration typically lasted for 1 h or a bit longer during the afternoon and evening hours in Los Angeles, California, and other large cities. This peak concentration was high enough to cause acute effects such as irritation to the eyes and the respiratory tract, lung function reduction, difficulty to breathe, and increased emergency room visits. However, this feature does not occur in most areas of the United States, because the regional transport of ozone precursors prolonged the hours of elevated ozone concentrations. Epidemiological studies have found that ozone concentrations averaged over a longer period (such as 8 h instead of 1 h) within a day are a more health-relevant indicator of ozone exposure. In fact, as of June 15, 2005, the 1-h ozone standard is no longer applied to areas designated with respect to the 8-h ozone standard, which includes most of the United States, except for portions of 10 states^2^.

Since the enaction of the U.S. Clean Air Act Amendment in 1970, remarkable efforts were made to control the emissions of the two ozone precursors (and other criteria pollutants). From 1980 to 2017, total national emissions of NO_X_ and VOCs were reduced by 61 and 54%, respectively. Consequently, there was a 32% decrease in national average of daily maximum 8-h averages of ozone measured at 200 monitoring sites across the United States^3^. Despite this nationwide decrease and more drastic decrease in some ozone “hot spots” such as Los Angeles and Atlanta, Georgia^4^,^5^, 100.6 million people nationwide (or nearly one in every three people) lived in U.S. counties where ozone levels exceeded the NAAQS standard of 70 ppb in 2017 (In contrast, much fewer people, 58 million in total lived in counties where PM_2.5_, PM_10_, SO_2_, or lead exceeded the NAAQS)^3^. If the WHO-recommended 8-h limit of 50 ppb is used, there would be even more people living in places with ozone exceeding the health-based limit.

Although ambient ozone concentrations have showed a declining trend in the United States and similarly in Western Europe and Japan in the past decades^6^, evidence suggests that global average ozone concentrations are increasing. For example, ozone measured at Mt Waliguan Observatory (a global “background” site) on the Tibetan Plateau over the period of 1994–2013 has shown an increasing trend at 0.2–0.3 ppb per year during spring and autumn (11). The springtime ozone increase was partly (~60%) attributed to increased stratosphere-to-troposphere transport, whereas rising Asian anthropogenic emissions of ozone precursors were the key driver of increasing autumnal ozone at this site. This finding is alarming, because ozone is generally considered too reactive to be transported afar. However, this demonstrates that NO_x_ and VOCs emitted in more populous regions can undergo long-range transport and affect “background” ozone level. More importantly, this finding also suggests an increasing ozone trend not only in places where NO_x_ and VOCs were originally emitted but also along the air trajectories from precursor sources to the background site.

Although the air quality focus has been on particulate matter, especially PM_2.5_, in rapidly developing economies such as China and India in the recent years, the “invisible” ozone pollution is increasingly recognized as a major health hazard. While annual PM_2.5_ average concentrations showed a decreasing trend in many cities of China (12), ground-level ozone concentrations measured in some monitoring sites in China showed an increasing trend in the past several years. For example, observations made at a rural site (Dadianzi) 100 km northeast Beijing showed a steady increase in annual averages of the 8-h daily max ozone concentrations from 2004 to 2015 (13). Consistently, a recent analysis of ground-level ozone concentrations measured at nearly 1,000 sites across China also found an increasing trend of summertime ozone in northeastern China from 2013 to 2017 (6). This increase was attributed to changes in anthropogenic emissions of ozone precursors as well as reductions in PM_2.5_ concentrations as described earlier. Due to the strong governmental efforts to primarily control PM_2.5_ in China, anthropogenic emissions of NO_x_ have decreased substantially in most urban areas of China (a 21% nationwide reduction) from 2013 to 2017 (6). However, VOC emissions have remained relatively unchanged. Typically decreasing NO_x_ would increase ozone under VOC-limited conditions, which has been the case for many urban areas of China. All these explain that in summer months, daily air quality reports released to the public in recent years have often shown more days when the ozone standard was exceeded than when PM_2.5_ or other regulated pollutants exceeded the standards in many cities of China. This trend in China appears to follow the pattern of the United States where non-compliance to ozone standard has been more frequently observed in more places than that to PM_2.5_ or other pollutants.

## Human Exposure and Dosimetry

It is the fundamental principle of toxicology that “dose makes poison.” It is common in air pollution epidemiologic studies to use ambient concentrations as a proxy for exposure or, more strictly speaking, dose. This approach omits inter- and intra-person differences in breathing rate and does not consider concentration differences between indoor and outdoor environments. Among common or the criteria pollutants defined by the US EPA, ozone has unique characteristics that can lead to substantial errors for using ambient ozone concentration as a proxy for dose.

First, ozone is chemically reactive and can be more effectively scavenged by building surfaces. In airtight buildings with doors and windows closed, indoor ozone levels are typically smaller than 20% of outdoor levels. In contrast, for leaky buildings and for building with windows frequently open, indoor concentrations can reach >70% outdoor concentrations. Because typically people spend the majority of time indoors, using outdoor concentration as a surrogate for ozone exposure would lead to greater overestimation of exposure for people living or working in more airtight buildings than for those living in less airtight buildings. This systematic exposure assessment error was used to explain a difference in ozone effect estimates in U.S populations living in buildings with different indoor–outdoor air exchange rates^1^. Recent advancement in small and low-cost ozone sensors makes personal monitoring or indoor monitoring more affordable and feasible. More accurate ozone monitoring can be used in future studies of ozone epidemiology and can also aid data-based personal prevention actions.

Second, outdoor ozone concentrations exhibit a substantial seasonal variation in most of the places. This adds challenges to assess the health effect of long-term exposure in epidemiological studies. Unlike using annual averages for other pollutants such as PM_2.5_, warm-season averages have often been used (14, 15), assuming that the health risk associated with lower-level ozone in cold months is negligible. Accordingly, certain ozone control policies have been implemented only during photochemical smog months. In the United States, for example, gasoline is formulated with higher oxygen content (typically with increased fraction of ethanol) in warmer months to reduce VOC emissions that contribute to ozone formation. However, accumulating evidence suggests that there may not exist a threshold ozone concentration below which the risk is “zero.” Therefore, completely ignoring cold months in ozone control strategy may need to be revisited.

Third, outdoor ozone typically exhibits a distinct diurnal pattern with high concentrations during afternoon and early evening hours. Hourly concentrations of ozone are usually reported at ambient monitoring stations. For regulatory purposes, these hourly data are computed as moving averages to identify maximum 1- and 8-h concentrations (based on moving averages) within a day. In epidemiological studies, using concentrations with different averaging times has different toxicological assumptions. Using 1-h max concentration is to assess the acute effect of peak exposure, whereas using 8-h daily max concentration assumes that lower concentrations during the rest of 16 h do not contribute to an adverse effect. It is also possible that either 1- or 8-h max concentrations were simply used due to the data availability in previous epidemiologic studies of short-term ozone effects (16–20). In addressing exposure to low-level concentrations (such as concentrations below the current air quality standard), however, 24-h average may be another relevant measure of daily exposure, at least for certain outcomes (21–24).

## Health Effects Evidence to Support Ozone Regulations

Following a formal process of an extensive literature review and a critical analysis, the US EPA summarized its evaluation of available evidence in the 2013 US EPA Integrated Science Assessment for Ozone^7^. Based on this assessment, the national ambient air quality standard for 8-h daily max ozone was revised from 75 to 70 ppb in 2015. The health effects evidence used to support these revisions include mainly the following, which has been well-demonstrated in recent reviews (25, 26).

- Ozone can cause adverse respiratory effects such as difficulty of breathing (e.g., shortness of breath and pain when taking a deep breath) and inflammation of the airways in the general population. These effects can aggravate lung diseases such as asthma, emphysema, and chronic bronchitis [chronic obstructive pulmonary disease (COPD)].
- Long-term exposure to ozone is likely to be one of many causes of asthma development.
- Ozone exposure is likely to cause premature deaths, and the evidence is stronger for mortality due to respiratory illnesses than for that due to other diseases.
- Children are at increased risk from ozone exposure, as children have a relatively higher dose per body mass and children's lung is still developing.

Does this revised standard imply that the effects listed above would not occur when 8-h daily max ozone concentrations are below 70 ppb? Although from a regulatory standard point, the public may be informed that it is “safe” to breathe the air when air quality meets the standards, it is easy to see that the standards are set somewhat arbitrary. For example, the WHO guideline for 8-h daily max ozone of 100 μg/m^3^ (~50 ppb) is lower than the EPA standard, but the evidence to support this lower limit is similar to that in supporting a higher limit by the US EPA, with additional effects presented by the WHO as follows^1^:

- Ozone can cause coughing and sore or scratchy throat.
- Ozone exposure makes the lungs more susceptible to infection.
- Ozone continues to damage the lungs even when the symptoms have disappeared.

Although WHO also considers that ozone is a cause of COPD, this evidence was not strong enough in the 2013 EPA integrated science assessment. Other effects of ozone reported include the following. On high ozone days, there have been increased school absences, increased visits to emergency rooms, and increased hospital admissions (27–30). Long-term exposures to ozone have been associated with lower lung function and deteriorated or abnormal lung development in children (31, 32). In both the WHO guideline and the EPA ozone standard, more susceptible populations are considered. In addition to people with preexisting respiratory diseases such as asthma and COPD, children, older adults, and people who are active outdoors (especially outdoor workers) are more vulnerable to ozone exposure.

## Immune-inflammatory Responses and Emerging Effects

As a potent oxidizing gas, ambient ozone is well-known to cause oxidative damages to the cells and the lining fluids of the airways, thereby inducing immune-inflammatory responses in the lung. Recent findings have shown that innate immunity is implicated in ozone-induced airway inflammation, such as the involvement of innate lymphoid cells (ILCs) in mice (33, 34). Ozone exposure contributes to the increased expression of mRNA of tumor necrosis factor-α (TNF-α), interleukin-1β (IL-lβ), interleukin-6 (IL-6), and interleukin-8 (IL-8) in human alveolar macrophages (35) and increased concentrations of IL-6, IL-8, and fibrinogenic proteins in human airway epithelial cells (36). A seminal work by Koren et al. demonstrated that an acute exposure to ozone (0.4 ppm for 2 h) resulted in 8.2-fold increase of polymorphonuclear leukocytes (PMN) in bronchial alveolar lavage (BAL) fluid and enhanced level of inflammatory mediators in the lower airways of humans (37). Krishna et al. further confirmed that ozone-induced neutrophil influx in human peripheral airways was partly mediated by IL-8 (38). Ozone exposure resulted in significant neutrophilic inflammation, reflected with increased levels of myeloperoxidase (MPO) in the supernatant of induced sputum samples from healthy subjects (39, 40).

These immune-inflammatory responses to ozone may “spill over” to the circulatory system, which may help explain emerging evidence on the cardiovascular and neuronal effects of ozone. Since the 2013 EPA assessment was released, several studies conducted in North America further confirmed significant positive associations, robust to controlling for co-pollutants, between short-term ozone exposure and one or more of the following mortality classifications: cardiovascular, dysrhythmia, cardiometabolic, and ischemic heart disease. A meta-analysis of 53 studies showed a weak but significant association between ozone and hospital admission and mortality from stroke (41). Significant associations of ozone were found with ischemic stroke occurrence in Seoul (42) and with non-myocardial infarction out-of-hospital cardiac arrests in Helsinki (43). Although Jerrett et al. (44) in the original analysis of an American Cancer Society cohort found that ozone exposure was associated with respiratory but not cardiovascular mortality, in the follow-up study using the same cohort, Turner et al. (14) found a significant association of long-term exposure to ozone with cardiovascular mortality. A recent cohort study by Lim et al. further confirmed this association between long-term exposure to ozone and increased cardiovascular mortality (45). However, there have also been epidemiological studies reporting null findings between long-term ozone exposure and cardiovascular mortality in Europe (46, 47).

Initiated in the lung, the immune-inflammatory responses to ozone may ultimately contribute to increased cardiovascular mortality and morbidity via two major pathways affecting hemostasis and autonomic tone. Increased exposure to ambient ozone has been associated with increased levels of hemostatic markers, including fibrinogen (48–50), von Willebrand factor (49), and plasminogen activator inhibitor-1 (48). Xia et al. revealed that short-term exposure to ambient ozone can elevate serum levels of ACE and ET-1, decrease their DNA methylation, and alter the lipid metabolism, which may be partly responsible for increased blood pressure and vascular endothelial disfunction (51). Day et al. found that an increase in 24-h or 2-week average exposure to ozone was associated with increased p-selectin (a soluble plasma marker of platelet activation), suggesting that ozone exposure increases the risk of thrombosis (21). Wang et al. found that increased ambient ozone exposure was associated with increased rate of carotid wall thickness progression and risk of new plaque formation in healthy adults (52). Jia et al. showed that ambient ozone exposure within several minutes can decrease heart rate variability in the healthy elderly subjects, suggesting that a dysfunction of cardiac autonomic nervous system may be involved (53). In controlled human exposure studies, a few hours of ozone exposure resulted in changes in markers of inflammation and fibrinolysis at 300 ppb and changes in cardiac autonomic function at 110–300 ppb (54, 55). Although one study found a blunting of exercise-induced blood pressure increases (56) and another found increased systolic blood pressure in response to ozone exposure (57), other such studies found increases in diastolic blood pressure to a co-exposure of ozone and concentrated ambient PM but not to ozone alone (58, 59). In contrast, animal studies with high ozone exposures have generated more consistent findings on cardiovascular effects of ozone through altering vascular tone (60–62), mRNA for genes encoding thrombogenic factors (63), and atherogenesis (60).

Additionally, deleterious effects of ozone exposure on the central nervous system (CNS) are emerging (64, 65). Neurodegenerative disorders, such as Alzheimer's disease (AD) and Parkinson's disease, have been linked to ozone exposures in recent epidemiologic studies (66, 67). The following toxicological studies in rodents have demonstrated the CNS effects of ozone, shedding light on biological mechanisms to support the link between ozone exposure and outcomes related to CNS. Rodríguez et al. showed that ozone exposure resulted in the activation of apoptotic death in rat hippocampus mediated by endoplasmic reticulum stress (68). Bello-Medina et al. found that rats chronically exposed to ozone exhibited deficits in learning and memory loss associated with deafferentation in hippocampus-related neurons (69). Chronic exposure to low-dose ozone, on the other hand, could enhance systemic and hippocampal Th17/IL-17A immune responses, which may be partly responsible for neurodegenerative effects in rats (70).

## Discussion and Conclusions

Ozone pollution is a worldwide health hazard. In many parts of the world, as described above, ozone concentrations are projected to increase, leading to increases in ozone-associated mortalities and morbidities. A study reported a 6% increase in premature deaths attributable to ozone globally from 1990 to 2010 (71), although the estimates (143,000 deaths in 1990 and 152,000 deaths in 2010) seem to be substantially lower than the estimated reported in other studies. In one study, for example, anthropogenic ozone was associated with an estimated 700,000 ± 300,000 respiratory mortalities in 2000 (72). In another study, exposure to ozone was responsible for 254,000 deaths from COPD alone in 2015 (73). Other ozone-associated mortality estimates include 316,000 respiratory deaths in China (15) and ~23,500 in the European Union^8^. The relatively large gap in the estimates across studies is due to uncertainties associated with and inconsistence in concentration–effect relationship and ozone exposure assessment. These estimates were based on respiratory effects alone, due to uncertainties associated with the current evidence on the cardiovascular effects. It is expected that the impact would be larger when other ozone effects were considered.

Ozone exposure was associated with large morbidity estimates. An estimated 9–23 million (8–20% of total) asthma-related emergency room visits globally were attributable to ozone (74). A large multicity study in China showed that short-term exposure to ambient ozone was associated with higher non-accidental and cardiovascular mortality (20). In addition, an estimated 23.0–40.3 million respiratory-related deaths were attributable to long-term O_3_ exposure in 2016 (15). In the European Union, ozone in 2010 was responsible for 19,200 cases of respiratory hospital admissions, 86,000 cases of cardiovascular hospital admissions, and over 109,000,000 minor restricted activity days^7^. Disability adjust life years (DALY) lost attributable to ozone were estimated to be 6.3 ± 3.0 million years in 2000 (58) and 4.1 (95% CI: 1·6–6·8) million years from COPD alone in 2015 (73).

Disease burden attributable to ozone is expected to continue to rise in the future for two reasons. The first is the fact that ozone concentration is on the rise in many parts of the world as described earlier. For example, an estimated increase of 0.43 ppb in average ozone concentration, during the 2040s compared to 2000 due to climate change alone, would correspond to a 0.01% increase in mortality rate in 19 urban communities in southeastern United States (9). Relative to that in 2000, there will be a 14% increase in global ozone-related mortality (75). The second is anticipated improvement in our understanding of the ozone effects beyond the lung and improved characterization of the chronic effects of long-term exposure. The improved knowledge will likely add to ozone-associated disease burden that is currently uncounted for.

There remain significant challenges in ascertaining chronic ozone exposure and effects that are currently inconclusive. Epidemiological evidence is limited to support a causal relationship between the chronic exposure and mortality or morbidity, although progresses have been made in recent years by using large datasets. For example, an analysis of national databases found a positive association between increase in long-term ozone concentrations and an increased risk of respiratory diseases and death (44). Di et al. (76) analyzed the entire US Medicare population of 60 million older adults from the years 2000 through 2012 and found a positive association between annual averages of ozone and all-cause mortality rate. Using large hospital records, increased chronic ozone exposure was associated with increased asthma hospital admissions in children (27). Long-term ozone exposure (3-year averages) has been associated with development of acute respiratory distress syndrome (ARDS) in at-risk critically ill patients, particularly in trauma patients and current smokers (77). By examining life expectancy at birth in 3,109 counties of the conterminous U.S. during 2002 to 2008 in relation to county-specific mean levels and rates of change in ozone concentrations, a study found that a 5 ppb (10 μg/m^3^) increase in long-term ozone concentration was associated with 0.25 year (95% CI: −0.30 to −0.19) lower life expectancy in males and 0.21 year (95% CI: −0.25 to −0.17) in females (78).

One of the challenges is to determine what is the best measure for long-term ozone exposure, given that ozone has distinct diurnal, and seasonal variations. A relevant question is whether repeated episodes of short-term high-level exposures can result in lasting health effects beyond the observed acute effects. Answering this question is not easy as some of the acute ozone effects are known to be reversible. What remains unknown is how much of the acute effects can be repaired or reversed between the episodes. At the meantime, people are constantly exposed to other air pollutants such as PM_2.5_. The co-exposure may affect the ability to repair the damage caused by acute ozone exposures. The natural fluctuation in ambient ozone concentration hence makes it challenging to examine chronic effects of short-term and long-term exposures. However, it is imperative to address such challenges in future studies of novel study design incorporating promising technologies in monitoring and computing ozone exposures with unprecedented accuracy and precision.

The large disease burden resulting from ozone pollution warrants persistent calls for control polices worldwide, but this is more urgent in developing countries where most of the attention is paid toward PM_2.5_ reductions. Based on the history of ozone control in the United States, aggregative regulatory actions to cut down anthropogenic emissions of NO_x_ and VOCs have not necessarily resulted in sufficient reductions in ozone concentrations in certain areas of the United States. Part of the challenges is the “non-linear” relationship of ozone production with its precursors, as reducing one of the precursors may not necessarily lead to ozone reduction. Even within a metropolitan area, the optimal ratio of NO_x_ to VOCs associated with minimal ozone formation changes from day to day (even hour to hour) and from upwind to downwind. Sources of VOCs can be numerous and hard to characterize. Some known sources, such as household use of consumer chemicals and biogenic emissions, are difficult to control through regulatory actions. Although the fundamental chemistry of ozone formation is clear, ozone concentrations, and spatiotemporal distributions are specific to local meteorological conditions, local sources of NO_x_ and VOCs, and long-range transport of ozone and associated chemical species. For all these challenges, ozone pollution in developing countries is expected to be a long-term problem, and local and national polices should be developed or strengthened to persistently combat ozone pollution.

In the United States and developed countries with relatively better air quality, following decades of controls for NO_x_ and VOCs emissions, further controls of anthropogenic emissions via policy, and technological tools are becoming increasingly limited. Meanwhile, predictions of ozone levels in response to changing NO_x_ and VOC concentrations get harder with ozone concentrations approaching their “background” level. Yet, emerging evidence does not support a threshold for adverse effects of ozone (79, 80). Or if a threshold exists, it would have to be substantially lower than the current health-based regulatory standards or guidelines.

Considering all these challenges, it is imperative to use other means to reduce the health impact of ozone. During high ozone hours, the public, especially children and those with preexisting health problems, is advised to avoid outdoor activities. Schools may be advised to cancel outdoor sports activities. Because indoor ozone levels are a small fraction of outdoor levels in airtight buildings with door/windows closed, this strategy can effectively reduce individuals' exposure to ozone. To further reduce outdoor exposure, individuals may consider wearing a face mask that can effectively scavenge ozone. Face masks rated N95 or higher can filter out PM_2.5_ effectively and are widely available in the market worldwide. However, few models of face masks are designed to remove ozone. Making ozone forecasting available to the general public will enhance the effectiveness of such personal protection methods to reduce ozone exposure (81).

A wealth of data from animal studies and human studies are available in the literature to help understand pathophysiologic mechanisms by which ozone affects the lung. Relatively less is known to understand how ozone affects the cardiovascular health outcomes, although the immune-inflammatory responses initiated in the lung are thought to be the key in more downstream systemic effects. The mechanistic understanding appears to be sufficient to support the use of antioxidants or ozone scavenger to alleviate the ozone effects. For example, rodent studies confirmed that the use of N-acetylcysteine and sulfide salt can help prevent or recover the lung impairment caused by ozone (82, 83). Limited studies in humans have shown promising results. A randomized trial found that a daily supplement of vitamins C and E might provide some protection against acute nasal inflammatory response to ozone in asthmatic children (84). In a control human exposure study (2-h exposure to 400 ppb ozone vs. filtered air), healthy adults who had received a dietary antioxidant supplementation of a mixture of vitamin C, alpha-tocopherol, and vegetable cocktail exhibited a significantly smaller ozone-induced reduction in pulmonary function (85). Cohort and population-based interventional trials should be conducted in real-world settings to develop more targeted preventive or therapeutic strategies especially in vulnerable populations and individuals. This should be part of the overall strategy, along with air pollution control polices, to combat ozone pollution, a lasting worldwide health hazard.

## Author Contributions

JZ and YW: conception. JZ, YW, and ZF: drafting the manuscript, editing and revising the manuscript. All authors gave final approval for publishing.

### Conflict of Interest

The authors declare that the research was conducted in the absence of any commercial or financial relationships that could be construed as a potential conflict of interest.

## Footnotes

^1^https://www.who.int/news-room/fact-sheets/detail/ambient-(outdoor)-air-quality-and-health

^2^https://www.epa.gov/criteria-air-pollutants/naaqs-table

^3^https://www.epa.gov/air-trends/ozone-trends

^4^https://calepa.ca.gov/

^5^https://www.epa.gov/ga

^6^http://www.futureearth.org/blog/2018-feb-5/powerful-new-dataset-reveals-patterns-global-ozone-pollution

^7^https://cfpub.epa.gov/ncea/isa/recordisplay.cfm?deid=247492

^8^http://ec.europa.eu/environment/air/pdf/TSAP%20CBA.pdf
